# Significant Enrichment and Diversity of the Staphylococcal Arginine Catabolic Mobile Element ACME in *Staphylococcus epidermidis* Isolates From Subgingival Peri-implantitis Sites and Periodontal Pockets

**DOI:** 10.3389/fmicb.2018.01558

**Published:** 2018-07-12

**Authors:** Aoife M. O’Connor, Brenda A. McManus, Peter M. Kinnevey, Gráinne I. Brennan, Tanya E. Fleming, Phillipa J. Cashin, Michael O’Sullivan, Ioannis Polyzois, David C. Coleman

**Affiliations:** ^1^Microbiology Research Unit, Division of Oral Biosciences, Dublin Dental University Hospital, University of Dublin, Trinity College Dublin, Dublin, Ireland; ^2^National MRSA Reference Laboratory, St. James’s Hospital, Dublin, Ireland; ^3^Division of Restorative Dentistry and Periodontology, Dublin Dental University Hospital, University of Dublin, Trinity College Dublin, Dublin, Ireland

**Keywords:** ACME, *Staphylococcus epidermidis*, periodontal disease, peri-implantitis, subgingival sites, oral cavity, periodontal pockets, *kdp* operon

## Abstract

*Staphylococcus aureus* and *Staphylococcus epidermidis* are frequent commensals of the nares and skin and are considered transient oral residents. Reports on their prevalence in the oral cavity, periodontal pockets and subgingivally around infected oral implants are conflicting, largely due to methodological limitations. The prevalence of these species in the oral cavities, periodontal pockets and subgingival sites of orally healthy individuals with/without implants and in patients with periodontal disease or infected implants (peri-implantitis) was investigated using selective chromogenic agar and matrix-assisted laser desorption/ionization time-of-flight mass spectrometry. *Staphylococcus epidermidis* was predominant in all participant groups investigated. Its prevalence was significantly higher (*P* = 0.0189) in periodontal pockets (30%) than subgingival sites of healthy individuals (7.8%), and in subgingival peri-implantitis sites (51.7%) versus subgingival sites around non-infected implants (16.1%) (*P* = 0.0057). In contrast*, S. aureus* was recovered from subgingival sites of 0-12.9% of the participant groups, but not from periodontal pockets. The arginine catabolic mobile element (ACME), thought to enhance colonization and survival of *S. aureus*, was detected in 100/179 *S. epidermidis* and 0/83 *S. aureus* isolates screened using multiplex PCR and DNA microarray profiling. Five distinct ACME types, including the recently described types IV and V (I; 14, II; 60, III; 10, IV; 15, V; 1) were identified. ACME-positive *S. epidermidis* were significantly (*P* = 0.0369) more prevalent in subgingival peri-implantitis sites (37.9%) than subgingival sites around non-infected implants (12.9%) and also in periodontal pockets (25%) compared to subgingival sites of healthy individuals (4.7%) (*P* = 0.0167). To investigate the genetic diversity of ACME, 35 isolates, representative of patient groups, sample sites and ACME types underwent whole genome sequencing from which multilocus sequence types (STs) were identified. Sequencing data permitted ACME types II and IV to be subdivided into subtypes IIa-c and IVa-b, respectively, based on distinct flanking direct repeat sequences. Distinct ACME types were commonly associated with specific STs, rather than health/disease states or recovery sites, suggesting that ACME types/subtypes originated amongst specific *S. epidermidis* lineages. Ninety of the ACME-positive isolates encoded the ACME-*arc* operon, which likely contributes to oral *S. epidermidis* survival in the nutrient poor, semi-anaerobic, acidic and inflammatory conditions present in periodontal disease and peri-implantitis.

## Introduction

*Staphylococcus aureus* and *Staphylococcus epidermidis* are common commensals of human skin and the nares and are highly proficient at forming biofilms. Both species are significant causes of nosocomial infections associated with indwelling medical devices (Song et al., 2013). It is now widely acknowledged that many of the antimicrobial resistance genes identified in clinical isolates of *S. aureus* were acquired from coagulase negative staphylococci (CoNS) such as *S. epidermidis* by transfer of mobile genetic elements (MGEs) (Otto, 2013). One very significant example of this is the horizontal acquisition by *S. aureus* of staphylococcal cassette chromosome-*mec* (SCC*mec*) elements harboring the methicillin resistance gene *mec* from *S. epidermidis*. To date, 13 different types of SCC*mec* (I-XIII) have been characterized in methicillin-resistant *S. aureus* (MRSA)^1^ differentiated according to the various combinations of *mec* and cassette chromosome recombinase (*ccr*) gene complexes present (International Working Group on the Classification of Staphylococcal Cassette Chromosome Elements, 2009; Wu et al., 2015; Baig et al., 2018). Many more SCC*mec* variants have been described in species in which methicillin-resistance (MR) is more common, such as *S. epidermidis* (MRSE) and other CoNS (Shore and Coleman, 2013).

The staphylococcal arginine catabolic mobile element (ACME) plays a role in colonization of human skin and evasion of host immune responses (Diep et al., 2008; Planet et al., 2013). It is considered an SCC-like element as, like SCC*mec*, it is flanked by homologous inverted and direct repeat sequences (DRs) that integrate into the same *attB* attachment site in the chromosomal *orfX* locus as SCC*mec* (Ito et al., 2001; Diep et al., 2006). Specific clonal lineages of *S. aureus* are known to harbor ACME, most notably the highly successful USA300 clone, which harbors a composite island (CI) composed of SCC*mec* type IVa and ACME type I (Diep et al., 2006). The prevalence of ACME has also been reported to be high in sequence type (ST) 239 MRSA isolates (43.7%) from screening swabs of hospitalized patients in Singapore (Hon et al., 2014) and in ST239-like (as determined by pulsed-field gel electrophoresis) bloodstream MRSA isolates (39%) recovered in Australia (Espedido et al., 2012).

Like SCC*mec*, ACME is more prevalent and exhibits greater diversity in *S. epidermidis*. Many studies have identified ACME in multiple STs of the predominant *S. epidermidis* clonal lineages based on multilocus sequence typing (MLST), suggesting that ACME originated in this species (Miragaia et al., 2009; Barbier et al., 2011; Onishi et al., 2013). To date, ACME has been detected in the range of 45.8–67.9% in *S. epidermidis* isolates recovered from disparate geographical locations, as well as in carriage and disease isolates (Diep et al., 2006; Miragaia et al., 2009; Barbier et al., 2011; Onishi et al., 2013).

The ACME genetic island ranges between 30 and 55 kb in size and is associated with three main gene clusters, the *arc* operon composed of the *arcR/A/D/B/C* genes, the *opp3* operon composed of the *opp3A/B/C/D/E* genes, and the recently revealed *kdp* operon, composed of the *kdpE/D/A/B/C* genes (Diep et al., 2006; O’Connor et al., 2018). These gene clusters encode an arginine deaminase pathway, an oligopeptide permease ABC transporter and a potassium ABC transporter, respectively. These three operons which can be present in ACME are in addition to the native chromosomal *arc*, *opp1* and *opp2*, and *kdp* operons in *S. aureus* (Diep et al., 2008; Xue et al., 2011; Price-Whelan et al., 2013).

Five distinct ACME types have been described to date, according to the presence of the *arc* and *opp3* operons (type I), the *arc* operon only (type II), the *opp3* operon only (type III), the *arc* and *kdp* operons (type IV), and all three *arc*, *opp* and *kdp* operons (type V) (Gill et al., 2005; Diep et al., 2006; Shore et al., 2011; McManus et al., 2017; O’Connor et al., 2018). Furthermore, two distinct ACME IV subtypes, IVa and IVb have been described based on distinct combinations of flanking DRs (O’Connor et al., 2018). To date, all five types and several variants thereof have been described in *S. epidermidis* (Miragaia et al., 2009; Barbier et al., 2011; Onishi et al., 2013; Soroush et al., 2016; McManus et al., 2017; O’Connor et al., 2018). In contrast, types I and II and variants thereof have been detected in *S. aureus*, commonly collocated with other genetic elements such as SCC*mec* or SCC-associated genes in CIs and separated from these adjacent elements by DRs (Diep et al., 2006; Shore et al., 2011; Kawaguchiya et al., 2013; Rolo et al., 2012).

Although staphylococci are considered transient members of the oral microflora, these species are prevalent in the oral cavities of the elderly and in people with dental infections such as periodontal disease (Murdoch et al., 2004; Friedlander, 2010). Periodontal disease is an inflammatory condition that can progress from gingivitis in response to dental plaque and affects the gingiva as well as the supporting periodontal structures (Hajishengallis, 2015). As periodontal disease progresses, enlargement of the gingival crevice occurs and leads to eventual detachment of the gingival tissue from the tooth resulting in periodontal pocket formation. Periodontal pockets provide a semi-anaerobic nutrient-poor environment that is ideal for plaque accumulation by resident oral microflora and is prone to decreases in pH resulting from physiological processes such as tissue repair (Percival et al., 2014).

The titanium-based oral implant can act as an ideal substrate for staphylococcal-based biofilm formation (Thurnheer and Belibasakis, 2016), as can the oxygen-depleted environment of periodontal and peri-implantitis pockets. Dental implants are indwelling medical devices made of titanium-based alloys that are placed in the bone of the mandible or maxilla to anchor a prosthetic crown, denture or bridge (Adell, 1981; Branemark et al., 1983). They consist of a shaft that is placed directly in the jaw bone and stabilized by subsequent osseointegration, and an abutment onto which a prosthesis is fitted. Similar to gingivitis, peri-implant mucositis is an inflammatory condition that affects the gingivae surrounding a dental implant, which can progress to peri-implantitis in which supporting bone surrounding an implant is gradually lost, potentially resulting in implant failure (Renvert and Polyzois, 2018).

Both of these oral diseases have a similar etiology in that they are both associated with dental plaque in which a shift from normal resident microflora to more periodontopathogenic species appears to occur (Nibali et al., 2015).

This study investigated the prevalence of *S. epidermidis* and *S. aureus* in the oral cavities, subgingival sites and periodontal pockets of patients with implants and natural teeth in states of both health and disease. Isolates recovered were investigated for ACME to determine if ACME could be a molecular marker for periodontal disease and/or peri-implantitis. Previous studies investigated the prevalence of ACME in both *S. aureus* and *S. epidermidis* in a range of carriage and infection sites (Miragaia et al., 2009; Barbier et al., 2011; Du et al., 2013; Onishi et al., 2013), however, to our knowledge, no studies have investigated the prevalence of ACME among oral staphylococcal isolates from periodontal pockets or peri-implantitis sites. A selection of the ACME-positive isolates identified in the present study were further investigated by whole-genome sequencing (WGS) in order to elucidate the genetic organization of the ACMEs in detail. Such investigations could yield important information regarding the potential genetic reservoir of ACME that exist among *S. epidermidis* for potential future spread to MRSA.

## Materials and Methods

### Study Group

Ethical approval for this study was granted by the St. James’s Hospital and Federated Dublin Voluntary Hospitals Joint Research Ethics Committee (JREC) and the Faculty of Health Sciences Ethics Committee of Trinity College Dublin, Ireland. Prior to enrollment in the study, all participants were provided with comprehensive patient information documentation and all participants included provided written consent. All documentation (including consent forms) provided to patients was pre-approved by the Research Ethics Committees.

All participants in the study met the following criteria: they were over 18 years of age, had a minimum of 10 natural teeth and were capable of providing informed consent. Participants were excluded from the study if they had any of the following factors: diabetes or asthma, pregnancy or lactation, blood-borne illnesses, steroid treatment within the year or antibiotics within 2 months prior to sampling. Patients with periodontal disease had a minimum of one periodontal site with a probing depth of greater than 6 mm and bleeding on probing (BOP). Patients with peri-implantitis were partially dentate and had one or more oral implants in place for a minimum of 5 years, at least one of which showed clinical signs of disease (Sanz and Chapple, 2012; Holtfreter et al., 2015). The study group consisted of 31 orally healthy patients with dental implants, 20 patients with periodontal disease, 21 patients with peri-implantitis and 64 orally healthy participants.

### Sample Collection and Processing

All clinical sampling was carried out by qualified Dentists at the Dublin Dental University Hospital (DDUH). Sub-gingival sites and periodontal pockets were sampled by inserting a PerioPaper^TM^ gingival fluid collection strip (Oroflow, Plainview, NY, United States) into the sub-gingival crevice or periodontal pocket for 30 s. Following sampling the collection strips were placed in sterile 2 ml screw-capped tubes (Sarstedt AG & Co., Numbrecht, Germany) containing 1 ml of nutrient broth (NB) (Oxoid Ltd., Hampshire, United Kingdom). In addition, oral rinse samples were collected by providing participants with sterile 100 ml plastic cups (Sarstedt AG & Co.) containing 25 ml sterile phosphate buffered saline (PBS) and instructing the participant to rinse their mouths with the PBS for 30 s before returning the rinse fluid to the same container. The anterior nares of participants were sampled using nitrogen-gassed VI-packed sterile transport swabs (Sarstedt AG & Co.). Following sampling, all samples were transported immediately to the microbiology laboratory and processed within 4 h. Vials containing PerioPaper^TM^ strips suspended in NB were vortexed at maximum speed for 1 min and 100 μl aliquots of the resulting cell suspension were plated onto mannitol salt agar (MSA) and Sa*Select*^TM^ chromogenic agar (Bio-Rad Laboratories, Hertfordshire, United Kingdom) agar. Oral rinse samples were processed by transferring a 1 ml aliquot to a sterile 1.5 ml Eppendorf Safe-lock^TM^ microfuge tube (Eppendorf, Hamburg, Germany) and centrifuged at 20,000 ×*g* for 1 min, after which the supernatant was discarded and the resultant pellet was resuspended in 200 μl NB. To isolate staphylococcal colonies, 100 μl aliquots of this cell suspension were plated on MSA and Sa*Select*^TM^. Nasal swabs were used to lawn the entire surface of MSA and Sa*Select*^TM^ plates. Inoculated MSA and Sa*Select*^TM^ plates were incubated at 37°C for 48 h in a static incubator (Gallenkamp, Leicester, United Kingdom).

### Culture, Identification and Storage of Isolates

Bacterial isolates were cultured on Columbia blood agar (Fannin Ltd., Dublin, Republic of Ireland) at 37°C for 48 h prior to identification by Vitek MS Matrix-Assisted Laser Desorption Ionization-Time of Flight Mass Spectrometry system (MALDI-TOF MS) (Vitek, bioMérieux Marcy l’Etoile, France) according to the manufacturer’s instructions. Multiple isolates were identified and stored from each sample for future analysis. All isolates were stored on Microbank^TM^ storage beads (Pro-Lab Diagnostics, Cheshire, United Kingdom) at -80°C.

### DNA Isolation and Detection of ACME by Multiplex PCR and DNA Microarray Profiling

Where possible, a minimum of two isolates were selected as representatives of each individual participant, distinct sample sites and each staphylococcal species recovered and screened by multiplex PCR to detect the presence of ACME.

Genomic DNA was extracted from isolates by enzymatic lysis using the buffers and solutions provided with the *S. aureus* Genotyping Kit 2 DNA microarray kit (Alere Technologies GmbH, Jena, Germany) and the DNeasy Blood and Tissue kit (Qiagen, Crawley, West Sussex, United Kingdom) according to the manufacturers’ instructions.

The presence of ACME was detected in isolates by multiplex PCR targeting the *arcA, opp3B* and *kdpA* genes harbored by ACME using the previously described *arcA*- and *opp3B*-directed primers (Diep et al., 2006; McManus et al., 2017) and incorporating primers targeting the *kdpA* gene (kdpF: 5′-CGGTTTAACTGGTGCGTT-3′ and kdpR: 5′-GCAATACATACAGCGTAGCC-3′) (O’Connor et al., 2018). PCR assays were carried out in 50 μl reaction volumes containing a 200 μM concentration of each deoxynucleoside triphosphate, 1.25 U of GoTaq polymerase (Promega, Madison, WI, United States), 10 μl (1×) of GoTaq FlexiBuffer (Promega), 2.5 μM MgCl_2_, 100 pmol of each primer, and 1 ng of the DNA template. Cycling conditions consisted of 94°C for 2 min, followed by 35 cycles of 94°C for 30 s, 60°C for 30 s, 72°C for 45 s and followed by a final elongation step of 72°C for 10 min. Amplification products (*arcA* product: 724 bp, *opp3B* product 530 bp, *kdpA* product: 241 bp) were separated by electrophoresis in 2% (w/v) agarose (Sigma-Aldrich Ltd., Wicklow, Republic of Ireland) containing 1X GelRed^®^ (Biotium Inc., Fremont, CA, United States) and visualized using an Alpha Innotech UV transilluminator (Protein Simple, San Jose, CA, United States).

The presence of *mec* and ACME-*arc* genes amongst *S. aureus* and *S. epidermidis* isolates investigated was also detected by DNA microarray profiling using the *S. aureus* Genotyping Kit 2.0 (Alere Technologies GmbH, Jena, Germany) according to the manufacturer’s instructions and as described previously (Monecke et al., 2008).

### Molecular Characterization of ACME Elements by WGS

A total of 35 *S. epidermidis* isolates selected as representatives of each patient group, sample site and ACME type present were subjected to WGS (**Table 1**). Libraries were prepared using Nextera XT library preparation reagents (Illumina, Eindhoven, Netherlands) and sequenced using an Illumina MiSeq desktop sequencer. For each isolate, reads were aligned with reference *S. epidermidis* and *S. aureus* genomes containing ACME and/or SCC elements downloaded from Genbank using a Burrows-Wheeler aligner (BWA) (Li and Durbin, 2009) to select the most appropriate reference ACME type to use as a scaffold. *De novo* assemblies were carried out on the reads for each ACME-harboring isolate using SPAdes version 3.6^2^. For each isolate, the reference genome that exhibited the highest degree of alignment with the relevant reads was used in a further alignment with the annotated contigs from the *de novo* assembly of the relevant isolate. Contigs identified as containing ACME- or SCC*mec*-associated DNA sequences were aligned, annotated and visualized using BioNumerics version 7.6 (Applied Maths, Sint-Martens-Latem, Belgium) and the Artemis sequence viewer (Berriman and Rutherford, 2003).

**Table 1:** Population of *Staphylococcus epidermidis* isolates subjected to whole genome sequencing.

Isolate	Sample site	Patient	ACME type/subtype	ACME/CI size (kb)	ST^a^	Genbank accession number^b^
**Participants with periodontal disease (*n* = 12)**
P8OR3	OR	P8	IVb	54.2	210	MG787414^b^
P9OR1	OR	P9	IIb	31.1	701	MH188462
P9PPH12	SG	P9	IIb	31.1	73	MH188463
P9PPHI1	SG	P9	IIb	31.1	73	MH188464
P11OR1	OR	P11	IIb	31.1	73	MH188465
P11PPH21	SG	P11	IIb	31.1	73	MH188466
P11PPP12	PP	P11	IIa	54.4	59	MH188467
P14OR1	OR	P14	I	54.3	17	MH188468
P14PPP2	PP	P14	IIc	53.9	672	MH188469
P14NS2	NS	P14	IIb	30.7	14	MH188470
P16OR1	OR	P16	III	53.9	329	MF346684^b^
P19PPP1	PP	P19	IIc	53.5	672	MH188471
**Participants with peri-implantitis (*n* = 9)**
PS36PD	SG	PS36	IVa	40.2	432	MG787422^b^
PS7OR	OR	PS7	IIb	32	73	MH188472
PS7P2	SG	PS7	IVa	67.8	153	MH188473
PS23P1	SG	PS23	IVb	54.2	153	MH188474
PS30PH	SG	PS30	IVa	68.3	153	MG787421^b^
PS34PI	SG	PS34	IIb	31.6	14	MH188475
PS8TI	SG	PS8	IIb	32	73	MH188476
PS19PH	SG	PS19	V	116.9	5	MG787423^b^
PS21NS	NS	PS21	IVa	55.8	297	MG787420^b^
**Orally healthy participants with implants (*n* = 6)**
I9OR1	OR	I9	IVa	67.8	153	MG787415^b^
I11OR1	OR	I11	III	45.1	329	MF346685^b^
I12OR1	OR	I12	I	39.9	7	MH188477
I14OR1	OR	I14	IVa	67.8	153	MG787416^b^
I23OR2	OR	I23	IIb	48	89	MH188478
I1PPP121	SG	I1	III	66.9	329	MH188479
**Orally healthy participants (*n* = 9)**
120PPC	SG	120	IVa	67.8	153	MG787417^b^
200OR2	OR	200	IIb	32	73	MH188480
201OR2	OR	201	IIa	27	59	MH188481
204OR1	OR	204	III	65.6	329	MF346683^b^
217PPP362	SG	217	IIa	74.6	59	MH188482
218PP361	SG	218	IVa	39	130	MG787418^b^
32BR	OR	32	IIb	32	73	MH188483
33BR	OR	33	IVa	48.8	17	MG787419^b^


*^*a*^The ST of each isolate was determined by uploading the sequence of seven housekeeping genes to the *S. epidermidis* MLST online database (https://pubmlst.org/sepidermidis/). ^*b*^The genetic structure of these ACMEs has been described previously (McManus et al., 2017; O’Connor et al., 2018). ACME, arginine catabolic mobile element; ST, sequence type; OR, oral rinse; PP, periodontal pocket; SG, subgingival site.*

In order to confirm the genetic organization and orientation of contigs, primers were designed using BioNumerics version 7.6 that targeted a minimum distance of 200 nucleotides from the contig boundaries. The target specificity of primers was confirmed using BLAST software^3^. All primers were supplied by Sigma–Aldrich Ltd. Contig gaps were closed based on PCR-based amplification and Sanger-based sequencing of these regions using the primers listed in Supplementary Table S1. Sanger-based sequencing was carried out commercially by Source BioScience (Waterford, Republic of Ireland).

Multiple alignments of the complete nucleotide sequences of the *arc*, *opp3* and *kdp* operons (including intragenic regions) from each isolate investigated were carried out using the Clustal Omega tool (Sievers et al., 2011). The nucleotide sequence from the first to last base of each operon, including any intragenic regions was compared among all isolates investigated in the present study.

### Determination of STs Among Isolates Subjected to WGS

The STs of ACME-harboring *S. epidermidis* isolates subjected to WGS were determined from the WGS data by examination of the nucleotide sequences of the loci used for the consensus *S. epidermidis* MLST scheme (Thomas et al., 2007). Briefly, the relevant nucleotide sequences were gleaned from the WGS data and inputted into the *S. epidermidis* MLST database online^4^ in order to define allelic profiles and STs.

### Statistical Analyses

In order to determine if the differences in the prevalence of staphylococcal species and isolates harboring ACME were significant between different sample sites or patient groups, two-tailed Fisher’s exact tests were utilized. These analyses were carried out using GraphPad QuickCalcs^5^. A *P* value of <0.05 was deemed statistically significant. Statistical power analyses were calculated using the DSS research statistical power calculator tool^6^ with a confidence interval of 5%.

### Nucleotide Accession Numbers

The Genbank database accession numbers for the nucleotide sequences of the *S. epidermidis* ACMEs previously characterized (McManus et al., 2017; O’Connor et al., 2018) and in the present study are listed in **Table 1**.

## Results

### Prevalence of *S. epidermidis* and *S. aureus* in the Oral Cavity

*Staphylococcus epidermidis* was recovered from the oral rinse samples of 18/20 (90%) patients with periodontal disease, 18/38 (47.4%) patients with peri-implantitis, 25/31 (80.6%) orally healthy patients with implants and 44/64 (68.8%) orally healthy participants (**Table 2**). *Staphylococcus epidermidis* was significantly more prevalent in the oral rinse samples of orally healthy patients with implants than in those with peri-implantitis (*P* = 0.0061, Power = 90%), however the difference in the prevalence of *S. epidermidis* in the oral rinse samples of patients with periodontal disease in comparison to orally healthy participants was not quite statistically significant (*P* = 0.081).

**Table 2:** Prevalence of ACME types harbored by *S. epidermidis* from distinct patient groups and anatomical sites.

Patients (*n*)	Sample site	Prevalence of *S. epidermidis* per patient (%)	Prevalence of *S. aureus* per patient (%)	Number of isolates harboring ACME (%)	Prevalence of *S. epidermidis* harboring ACME (%)	ACME types identified among *S. epidermidis* (*n* = patients)							

						I	II	III	IV	V	I and II	II and III	I and IV
Periodontal disease (20)^a^	OR	18/20 (90)	5/20 (25)	14/27 (51.9)	12/20 (60)	2	5	1	3	0	0	1	0
	PP	6/20 (30)^3^	0/20 (0)	6/9 (66.7)	5/20 (25)^7^	0	3	0	1	0	1	0	0
	SG	4/20 (20)	0/20 (0)	5/6 (83.3)	4/20 (20)	1	3	0	0	0	0	0	0
Peri-implantitis (29, 38)^b^	OR	18/38 (47.4)^1^	8/38 (21.1)^2^	4/15 (26.7)	4/38^c^ (10.5)	0	3	1	0	0	0	0	0
	SG	15/29 (51.7)^4^	3/29 (10.3)	13/19 (68.4)	11/29 (37.9)^6,8^	0	8	1	1	1	0	0	0
Healthy with implants (31)	OR	25/31 (80.6)^1^	15/31 (48.4)^2^	24/39 (61.5)	19/31 (61.3)^5^	3	8	2	3	0	3	0	0
	SG	5/31 (16.1)	4/31 (12.9)	5/9 (55.6)	4/31 (12.9)^8^	1	0	1	1	0	0	0	1
Orally healthy (64)	OR	44/64 (68.8)	19/64 (29.7)	26/50 (52)	23/64 (35.9)^5^	2	15	1	3	0	0	2	0
	SG	5/64 (7.8)^3,4^	5/64 (7.8)	3/5 (60)	3/64 (4.7)^6,7^	0	1	0	2	0	0	0	0

*Significant differences in the prevalence of *S. epidermidis* between paired sample types from distinct patient groups are indicated by identical numerals in superscript. ^*a*^Subgingival samples were taken from patients with periodontal disease prior to any dental treatment. ^*b*^Oral rinse samples were recovered from 38 patients with peri-implantitis; subgingival samples were taken from 29 of these patients. ^*c*^Isolates recovered from oral rinse samples of four patients could not be located to be screened for the presence of ACME and thus were not included in statistical analyses. OR, oral rinse; SG, subgingival site; PP, periodontal pocket.*

The prevalence of *S. aureus* was considerably lower than *S. epidermidis* among all four groups of participants examined, detected in 5/20 (25%) patients with periodontal disease, 8/38 (21.1%) patients with peri-implantitis, 15/31 (48.4%) orally healthy patients with implants and 19/64 (29.7%) of orally healthy participants (**Table 2**). The prevalence of *S. aureus* was highest in the oral cavities of healthy patients with oral implants and was significantly more prevalent in the oral rinse samples of this patient group when compared to the corresponding sample sets from patients with peri-implantitis (*P* = 0.0219, Power = 77.8%).

### Prevalence of *S. epidermidis* and *S. aureus* in Subgingival Sites, Peri-implant Sites and Periodontal Pockets

*Staphylococcus epidermidis* was recovered from the periodontal pockets of 6/20 (30%) patients with periodontal disease and the peri-implant sites of 15/29 (51.7%) patients with peri-implantitis. In contrast*, S. epidermidis* was only recovered from the subgingival sites of 5/31 (16.1%) orally healthy patients with implants and 5/64 (7.8%) of orally healthy participants (**Table 2**). *Staphylococcus epidermidis* was significantly more prevalent in the periodontal pockets of patients with periodontal disease than the subgingival sites of orally healthy participants (*P =* 0.0189, Power = 77.1%). Similarly, the prevalence of *S. epidermidis* was significantly higher in the subgingival sites of patients with peri-implantitis than similar sites in orally healthy patients with implants (*P =* 0.0057, Power = 91.4%).

*Staphylococcus aureus* was equally or less prevalent than *S. epidermidis* in the subgingival sites of all participant groups investigated, recovered from none of the periodontal pockets of patients with periodontal disease, 3/29 (10.3%) peri-implant pockets of patients with peri-implantitis, 4/31 (12.9%) of orally healthy patients with implants and 5/64 (7.8%) of orally healthy participants (**Table 2**). The prevalence of subgingival *S. aureus* was not significantly different in any of the four participant groups investigated.

### Prevalence of ACME Among *S. epidermidis* and *S. aureus* Isolates Recovered

The Arginine catabolic mobile element was detected in 100/179 (55.9%) of the *S. epidermidis* isolates recovered from all four participant groups (**Table 2**). The *mecA* gene was detected in 12/179 (6.7%) isolates; two from patients with periodontal disease, three from patients with peri-implantitis, three from orally healthy patients with implants and four from orally healthy participants. In total, 5/12 of the MRSE isolates identified also harbored ACMEs, predominantly type II.

Among the samples from which *S. epidermidis* was recovered, ACME was detected in isolates recovered from the oral rinse samples of 12/20 (60%) patients with periodontal disease, 4/38 (10.5%) patients with peri-implantitis, 19/31 (61.3%) orally healthy patients with implants, and 23/64 (35.9%) orally healthy participants. The prevalence of *S. epidermidis* isolates harboring ACME was significantly higher in the oral rinse samples of orally healthy patients with implants than in those of healthy participants (*P* = 0.0275, Power = 76.1%).

Although the prevalence of *S. epidermidis* was lower in subgingival sites than in the oral rinse samples of all four participant groups, the proportion of ACME-harboring *S. epidermidis* isolates was higher (**Table 2**). The presence of ACME was detected in *S. epidermidis* isolates from the periodontal pockets and subgingival sites of 5/20 (25%) and 4/20 (20%) patients with periodontal disease, respectively. Similarly, ACME was detected in 11/29 (37.9%), 4/31 (12.9%) and 3/64 (4.7%) *S. epidermidis* isolates from the subgingival sites of patients with peri-implantitis, healthy patients with dental implants and orally healthy participants, respectively.

Isolates harboring ACME were significantly more prevalent in subgingival samples of patients with peri-implantitis than in subgingival samples of orally healthy participants (*P* = 0.0001, Power = 98.4%). Similarly, isolates with ACME were also significantly more prevalent in periodontal pockets of patients with periodontal disease than subgingival sites of orally healthy participants (*P* = 0.0167, Power = 78.5%). Interestingly, the prevalence of ACME-harboring isolates was also significantly higher in the subgingival sites of patients with peri-implantitis than subgingival sites of orally healthy patients with implants (*P* = 0.0369, Power = 72.9%).

In contrast, ACME was not detected in any of the 83 *S. aureus* isolates recovered from oral rinse samples (*n* = 56) and subgingival samples (*n* = 27) investigated.

All five previously described ACME types were detected in *S. epidermidis* isolates in the present investigation by multiplex PCR. In all participant groups and anatomical sites sampled, ACME type II was the predominant ACME type, identified in *S. epidermidis* isolates recovered from 53 of the participants investigated. The recently described ACME types IV and V were detected in *S. epidermidis* isolates recovered from 15 and one participants sampled, respectively (**Table 2**).

Pairs of separate *S. epidermidis* isolates recovered from the oral rinse and subgingival samples of four orally healthy patients with implants were found to harbor distinct ACME types (i.e., types I and II or types I and IV). Similarly, a pair of *S. epidermidis* isolates recovered the same periodontal pocket of a patient with periodontal disease harbored ACME types I and II, respectively (**Table 2**). Furthermore, pairs of separate *S. epidermidis* isolates harboring ACMEs II and III were detected in the same oral rinse sample of one patient with periodontal disease and in two orally healthy participants.

A total of 35 *S. epidermidis* isolates selected as representatives of each ACME type, patient group, sample site and ACME type were subjected to WGS in order to elucidate the genetic organization of the ACMEs harbored in detail (**Table 1**).

### Genetic Diversity of ACME Type I

Two isolates recovered from the oral rinse samples of a patient with periodontal disease (P14OR1) and an orally healthy patient with implants (I12OR1) were found to harbor ACME type I by multiplex PCR and were subjected to WGS. The STs of these isolates were identified as ST17 and ST7, respectively (**Table 1**). These STs are double locus variants of each other, differing at the *gtr* and *pyrR* loci by a total of four bp.

**FIGURE 1:**
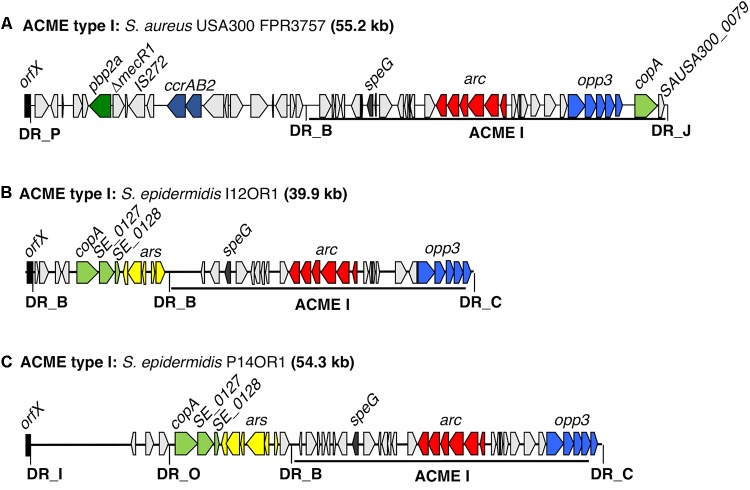
Schematic representation of ACME type I elements harbored by two distinct *S. epidermidis* isolates investigated by WGS. The ACME type I previously described in the MRSA reference USA300 strain FPR3757 (Genbank accession number CP000255.1) is included for comparison **(A)**. The size of each ACME is indicated after each strain name. **(B,C)** Each gene or group of genes of interest is differentiated by a different color, i.e., light blue; *opp3*-operon, red; *arc*-operon, pale green; *cop*-operon, yellow; *ars* operon, dark blue; *ccrAB* complexes, dark green; *pbp2a*, dark gray; *speG*, light gray; genes encoding hypothetical proteins, sugar transporters, transposases and other ORFs, previously identified in ACMEs. The direction of transcription for each ORF is indicated by arrows. The DRs are indicated in bold font and correspond to DR sequences listed in **Table 3**.

In both isolates, the ACME type I element was collocated with additional modules in CIs that were 54.3 and 39.9 kb in size, respectively (**Figure 1**). Both of the ACME type I elements characterized harbored the *arc* and *opp3* operons, the *speG* gene and were demarcated by DRs_B and _C (**Table 3**). In both isolates, ACME type I was located directly downstream of a module composed of the *copA* gene and the *ars* operon, separated by DR_B. In isolate I12OR1, this module was demarcated by an additional DR_B at the 5′ end in *orfX*, whereas in isolate P14OR1 this module was demarcated by DR_O at the 5′ end and an additional module containing three ORFs was located upstream of the *copA/ars* operon module (**Figure 1**).

**Table 3:** Direct repeat sequences (DRs) identified among ACME types investigated.

DR	Sequence (5′-3′)	Associated ACME types/subtypes and CIs
DR_A	GAAGCATATCATAAATGA	IIa, IIb, IIc, IVa, V
DR_B	GAAGCGTATCACAAATAA	I, IIa, III^a^, IVa, V
DR_C	GAAGCGTATCGTAAGTGA	I, IIa, IIb, III^a^, IVa, IVb, V
DR_D	GAAGCGTACCACAAATAA	IIa, IIb,
DR_E	GAAGCGTATTAAAGTGAT	IIc
DR_F	GAAAGTTATCATAAGTGA	IVb, V
DR_G	GAAGCGTATAATAAGTAA	IIa, IIb, III^a^, IVa, IVb, V
DR_H	GAAGCGTATCATAAGTGA	IIa
DR_I	GAAGCGTATCATAAATGA	I
DR_J	GAGGCGTATCATAAGTAA	I
DR_L	GAAGCATATCATAAGTGA	IIa, III^a^, V
DR_M	GAAGGGTATCATAAATAA	III^a^
DR_N	GAAGCGTATCACAAATGA	IVa
DR_O	GAAGCATATCATAAATAA	I
DR_P	GAAGCTTATCATAAATGA	I


*^*a*^The DRs _B, _L, _M, _G and _C described in the present study correspond to the DR1_A, DR_1B, DR_2, DR_3 and DR_4 previously described in ACME type III structures (McManus et al., 2017).*

### Genetic Diversity of ACME Type II

**FIGURE 2:**
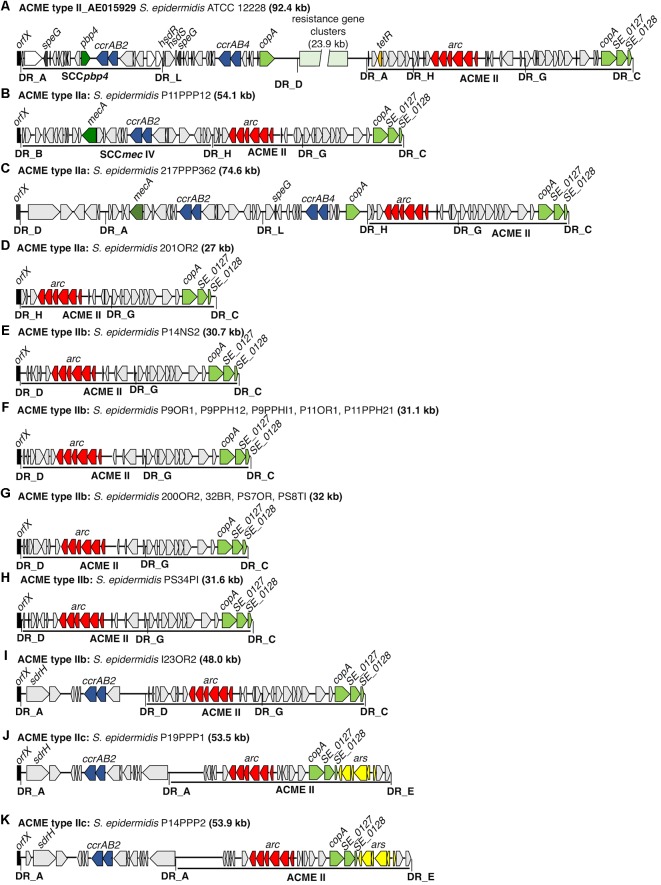
Schematic representation of ACME II subtypes a-c and ACME type II-harboring CIs investigated in the present study. The previously described ACME type II CI harbored by the *S. epidermidis* reference strain ATCC12228 (Genbank accession number AE015929) is included for reference **(A)**. The size of each ACME is shown after the strain name. Each gene or group of genes of interest is differentiated by a different color, i.e., red; *arc*-operon, pale green; mustard; *tetR*, *cop*-operon, yellow; *ars* operon, dark blue; *ccrAB* genes, dark green; *mecA*, dark gray; *speG*, light gray; genes encoding hypothetical proteins, sugar transporters, transposases and other ORFs, previously identified in ACMEs. The direction of transcription for each ORF is indicated by the arrow. The DRs are indicated in bold font and correspond to each DR sequence listed in **Table 3**.

Seventeen isolates harboring ACME type II were identified by multiplex PCR and were further investigated by WGS (**Figure 2**). All 17 isolates harbored ACMEs with the *arc* operon only and lacked both the *opp3* and *kdp* operons. Based on the differing combinations of DRs identified at the 5′ and 3′ ends, the ACME type II elements characterized could be divided into three distinct subtypes (IIa-c) of which ACME type IIb predominated (12/17, 70.6%). ACME type IIa was demarcated by the DRs _H and _C (**Table 3**) and was identified in 3/17 isolates (**Figures 2B–D**), all of which belonged to ST59. ACME type IIb (**Figures 2E–I**) was demarcated by the DRs _D and _C and was identified in 12/17 isolates belonging to ST89 (*n* = 1), ST14 (*n* = 2), ST73 (*n* = 8) and ST701, a single locus variant of ST73 (*n* = 1). The remaining two isolates harbored ACME type IIc demarcated by the DRs _A and _E and belonged to ST672 (**Figures 2J,K**). Interestingly in the case of the latter two isolates, the *copA* and *ars* operon were internalized within ACME type IIc in both isolates (**Figures 2J,K**) and both lacked the internal DR_G commonly identified downstream of the *arc* operon in ACME type II. The ACME type IIc-harboring CIs in these isolates were almost identical, differing only by the presence of an ORF upstream of the *sdrH* gene in isolate P14PPP2 (**Figure 2K**).

Five of the 17 ACME type II structures characterized by WGS existed as modules of larger CIs and were collocated with modules which harbored genes associated with SCC elements such as *ccr* and *mec* (**Figures 2B,C,I–K**). In each of these five CIs, the ACME type II structure was divided from these SCC-associated modules by DRs _A, _H, or _D (**Table 3**).

Interestingly, in one isolate (217PPP362) two separate and distinct SCC-associated modules were detected in tandem upstream of ACME type IIa (**Figure 2C**). The module immediately upstream of ACME type IIa was demarcated by the DRs _L and _H and harbored the *speG* and *ccrAB4* genes and was collocated immediately downstream of an additional SCC-associated module which harbored the *mecA* and *ccrAB2* genes. The ACME type IIa and IIb structures in the remaining 12/17 isolates investigated had integrated directly into *orfX* in the absence of any adjacent modules and were not components of larger CIs (**Figure 2**).

The presence of *sdr* genes, members of the serine/aspartate repeat family encoding fibrinogen-binding proteins was detected in modules adjacent to ACME type II in 3/17 isolates investigated and was collocated with ACME subtype IIb and IIc identified in the present study.

The *speG* gene encoding spermidine acetyltransferase was detected in only one isolate harboring ACME type II, (217PPP362) located in a SCC-associated module upstream of ACME type IIa (**Figure 2C**). The *copA* gene was located near the 3′ end of all 17 ACME type II structures investigated. In all ACME type IIa and IIb structures investigated, this gene was separated from the *arc* operon by DR_G and additional open reading frames commonly identified within ACMEs (**Figure 2**).

### Genetic Diversity of ACME Type III

**FIGURE 3:**
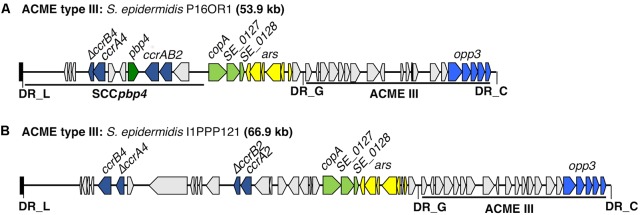
Schematic representation of ACME type III structure from oral *S. epidermidis* isolate I1PPP121 characterized in the present study. The previously described ACME type III structure from an *S. epidermidis* isolate (P16OR1) recovered from an oral rinse sample from a patient with periodontal disease (McManus et al., 2017) is included for comparison **(A)**. The size of the ACME characterized is shown after the isolate name I1PPP121 **(B)**. Each gene or group of genes of interest is differentiated by a different color, i.e., pale green; *cop*-operon, dark blue; *ccrAB* gene complexes (of which the genes *ccrA4* and *ccrB2* were prematurely truncated), light blue; *opp3* operon, dark green; *pbp4*, light gray; genes encoding hypothetical proteins, sugar transporters, transposases and other ORFs, previously identified in ACMEs. The direction of transcription for each ORF is indicated by the arrow. The DRs are indicated in bold font and correspond to each DR sequence listed in **Table 3**.

Four isolates harbored ACME type III, all of which were identified as ST329 (**Table 1**). Three of the ST329 isolates (P16OR1, 204OR1 and I11OR1) have been described previously (McManus et al., 2017). The CI harbored by the fourth isolate (I1PPP121) consisted of two distinct modules separated by DR_G (**Figure 3**). The module at the 5′ end harbored two pairs of *ccr* genes, of which *ccrA4* and *ccrB2* were prematurely truncated. This module also harbored the *copA* and *ars* genes, located upstream of ACME type III as previously observed in the other three isolates. The ACME type III harbored by I1PPP121 exhibited a minimum of 99.81% nucleotide identity to the ACME type IIIs harbored by isolates P16OR1, 204OR1, and I11OR1.

### Genetic Diversity of ACME Types IV and V

Eleven isolates harboring ACME type IV were identified and belonged to ST210 (*n* = 1), ST153 (*n* = 6), ST130 (*n* = 1), ST17 (*n* = 1), ST297 (*n* = 1), and ST432 (*n* = 1) (**Table 1**). Nine of these isolates were described recently in a study that first defined ACME types IV and V in *S. epidermidis* (O’Connor et al., 2018). The two additional ST153 isolates (PS7P2 and PS23P1) were characterized in the present study and harbored ACME types IVa and IVb, respectively. The ACME type IVa harbored by isolate PS7P2 was identical to those previously described in isolates 120PPC, I9OR1 and I14OR1, and the ACME type IVb harbored by isolate PS23P1 was identical to that previously described in isolate P8OR3 (O’Connor et al., 2018). ACME type V was identified in only one isolate, recovered from the subgingival site of a patient with peri-implantitis and has been previously described (O’Connor et al., 2018).

### Genetic Diversity Among the *arc*, *opp3* and *kdp* Operons Harbored by ACME Types I–V

#### The *arc* Operon

The percentage nucleotide identity between all ACME-*arc* operons identified in the present study, in ACME types I, II, IV and V ranged from 99.06 to 100%. The ACME-*arc* operons harbored by isolates 200OR2, 32BR, PS7OR and PS8TI (all ACME type IIb) exhibited 100% nucleotide identity to each other, as did that harbored by isolates 120PPC, I9OR1, I14OR1 (all ACME type IVa). Similarly, the ACME-*arc* operon harbored by P9OR1, P9PPH12, P9PPHI1, P11OR1 and P11PPH21 (all ACME type IIb) exhibited 100% nucleotide identity with each other (Supplementary Table 2).

#### The *opp3* Operon

The percentage nucleotide identity between all *opp3*-operons identified in the present study, in ACME types I, III and V ranged from 97.22 to 100% (Supplementary Table S3). The *opp3* operon harbored by isolates P16OR1 and P11OR1 (both ACME type III) exhibited 100% nucleotide identity to each other. The *opp3* operons harbored by ACME type I exhibited 99.91% to each other and 99.96% nucleotide homology to the *opp3* operon harbored by the reference ACME type I (Genbank accession number CP000255.1). The *opp3* operon harbored by the recently described ACME type V (O’Connor et al., 2018) exhibited 99.22-99.24% and 97.48–97.52% nucleotide identity to those harbored by ACME types III and I, respectively (Supplementary Table S3).

#### The *kdp* Operon

The *kdp* operon was highly conserved, exhibiting a minimum of 99.86% nucleotide identity amongst isolates harboring ACME types IV and V. The *kdp* operons harbored by isolates 120PPC, PS30PH, 33BR, 218PP361, PS7P2 and I9OR1 (all ACME type IVa) exhibited 100% nucleotide identity to each other, and to that harbored by PS23P1 (ACME type IVb). The *kdp* operon harbored by the remaining ACME type IVb isolates, PS36PD and P8OR3 exhibited 99.94% nucleotide identity to each other. The *kdp* operon harbored by ACME type V exhibited a minimum of 99.86% nucleotide identity to the *kdp* operon harbored by ACME types IVa and IVb (Supplementary Table S4).

### Comparison of ACMEs Among Multiple *S. epidermidis* Isolates From the Same Patients

Three isolates recovered from the periodontal pockets and oral rinse sample of a patient with periodontal disease (P9) were all identified as ST73 (**Table 1**) and harbored genetically identical ACME type IIb structures (Supplementary Figure S1). In contrast, three distinct *S. epidermidis* isolates recovered from the oral rinse, nasal swab and periodontal pocket of a patient (P14) with periodontal disease were identified as STs 17, 14 and 672, and harbored ACME types I, IIb and IIc, respectively (Supplementary Figure S1). Similarly, an isolate identified as ST59 and harboring ACME type IIa was recovered from the periodontal pocket of a patient with periodontal disease (P11) and was genetically distinct to two other isolates identified as ST73 and harboring ACME type IIb which were recovered from the oral rinse and another periodontal pocket of the same patient.

## Discussion

### The Prevalence of Staphylococci in the Oral Cavity, Subgingival Sites and Periodontal Pockets

Previous investigations of the prevalence of staphylococcal species in the oral cavity are conflicting and/or ambiguous for several probable reasons. Firstly, studies did not definitively distinguish between distinct CoNS species and *S. aureus* (Leonhardt et al., 1999). Secondly, different studies used semi-discriminatory agar media such as Baird Parker or MSA for primary recovery, which may have resulted in failure to select and further distinguish between morphologically similar colonies of distinct CoNS species (Loberto et al., 2004; Cuesta et al., 2010; dos Santos et al., 2014). Thirdly, several previous studies relied on checkerboard DNA-DNA hybridization techniques for definitive identification of oral staphylococcal species from patients with dental implants, an approach that does not distinguish between viable and dead bacteria (Fürst et al., 2007; Renvert et al., 2008; Salvi et al., 2008). Subsequently, real-time PCR with species-specific primers demonstrated that the previously used DNA:DNA hybridization probes showed cross reactivity between *S. aureus* and *S. epidermidis* DNA (Cashin, 2013).

Patient sampling and recovery of viable isolates was undertaken using a uniform, systematic methodology for all sample sites and patient groups and identification of both *S. epidermidis* and *S. aureus* isolates was determined using robust procedures. The utilization of the chromogenic Sa*Select*^TM^ medium for primary isolation of oral staphylococci enabled direct visualization and presumptive identification of both *S. epidermidis* and *S. aureus* isolates based on the growth of these species as white/pale pink and pink colonies, respectively. This approach ensured that the differences in the prevalence of *S. epidermidis* and *S. aureus* observed are a true reflection of the patient groups and sample sites investigated.

#### Staphylococcus epidermidis

*Staphylococcus epidermidis* was detected in the oral cavities of 47.4 – 90% of the four participant groups investigated (**Table 2**) which was higher than previous studies that reported its recovery from the oral cavities of 27.3% patients with periodontal disease (Loberto et al., 2004) and 41% of orally healthy participants (Ohara-Nemoto et al., 2008).

Previous reports of the prevalence of subgingival *S. epidermidis* vary greatly, ranging between 15.9 and 64.3% in periodontal pockets (Loberto et al., 2004; Murdoch et al., 2004; dos Santos et al., 2014) and between 42.9 and 60.7% in the subgingival sites of healthy participants (Murdoch et al., 2004; Ohara-Nemoto et al., 2008), most likely due to different methods used. Data on the subgingival prevalence of *S. epidermidis* in patients with oral implants and/or peri-implantitis are largely lacking or did not undertake definitive identification of this species (Leonhardt et al., 1999).

#### *Staphylococcus aureu*s

In the present study, *S. aureus* was considerably less prevalent than *S. epidermidis* in the oral cavities of participant groups and was rarely detected in subgingival sites or periodontal pockets (**Table 2**). The dramatic contrast in the oral prevalence of *S. epidermidis* and *S. aureus* was not surprising, as a negative correlation between the prevalence of these species has been reported previously (Brescó et al., 2017). Previous reports of the prevalence of subgingival *S. aureus* vary greatly, ranging from 13.4 to 68.2% (Cuesta et al., 2010; Zhuang et al., 2014) in periodontal pockets, 40–70.4% of peri-implant pockets and 25–44% of healthy subgingival sites around dental implants (Fürst et al., 2007; Renvert et al., 2008; Salvi et al., 2008). The contrasting results between the present and previous studies most likely reflects differences in methodology used as discussed above.

### The Prevalence of ACME in *S. epidermidis* and *S. aureus*

The prevalence of ACME has previously been investigated amongst *S. epidermidis* populations and has been reported to range from 40 to 65.4% in MRSE, and from 64.4 to 83% in methicillin susceptible *S. epidermidis* (Miragaia et al., 2009; Barbier et al., 2011; Du et al., 2013; Onishi et al., 2013). In the present investigation, only 12/179 (6.7%) of the *S. epidermidis* isolates recovered were MRSE. This finding correlates with previous studies that reported a higher prevalence of ACME in methicillin-susceptible *S. epidermidis*. Five of the 12 MRSE isolates identified here harbored ACMEs, predominantly ACME type II.

This is the first investigation into the prevalence of ACME amongst *S. epidermidis* and *S. aureus* isolates recovered from both above and below the gumline in patients with and without oral disease. The prevalence of ACME was greater than 50% amongst the populations of *S. epidermidis* recovered. In contrast, ACME was not detected in any of the *S. aureus* isolates recovered.

This study revealed for the first time that the prevalence of ACME-harboring *S. epidermidis* was significantly higher in subgingival sites of patients with peri-implantitis and periodontal disease in comparison to healthy individuals (*P* = 0.0001 and *P* = 0.0167, respectively). Furthermore, *S. epidermidis* harboring ACME were significantly more prevalent in the subgingival sites of patients with peri-implantitis than in orally healthy patients with implants. Together, these results suggest a strong association of *S. epidermidis* isolates harboring ACME with diseased, semi-anaerobic subgingival tissue sites. No correlation between specific ACME types or subtypes and specific disease state or oral site was identified.

One of the potential limitations of the present investigation was the number of patients sampled. The study group was limited to patients attending DDUH who did not have any underlying conditions and had not received antibiotics in the 2 months prior to sampling. It is likely that larger investigations would further support the findings of this study as well as likely identify additional ACME types. Furthermore, it would be interesting to determine if ACME is more abundant in *S. epidermidis* isolates recovered from other diseased anatomical sites or wounds.

### The Genetic Diversity of ACME

Previous investigations of the prevalence and structural diversity of ACME relied on primers targeting the ACME-*arc* and ACME-*opp3* genes to detect ACME types I-III only (Miragaia et al., 2009; Onishi et al., 2013). This study is the first to include primers targeting the recently described ACME-*kdp* operon in a multiplex ACME-typing PCR and therefore the results of this study accurately reflect the true prevalence of ACME types currently described in *S. epidermidis*, at least of oral origin. The application of WGS to the characterization of ACME has revealed the additional ACME types harbored by *S. epidermidis* in addition to the detailed structural diversity of these ACMEs. It is highly likely that *S. epidermidis* isolates harboring the *opp3* and *kdp* operons and the *kdp* operon only will also be identified in the future using WGS.

Common DRs were often observed amongst multiple ACME types and subtypes (**Table 3**). Many distinct DRs have been identified at the 5′ end of ACME, which is clearly demarcated by the integration of this element into *orfX*. The demarcation of the 3′ end is less obvious. Previously described ACMEs terminated at the 3′ end by DR_J in ACME type I of *S. aureus* (Genbank accession number CP000255.1) (Diep et al., 2006) and by DR_C in ACME types II-V of *S. epidermidis* (Gill et al., 2005; McManus et al., 2017; O’Connor et al., 2018), even though an internal DR_G is present downstream of the *arc* operon in both the reference ACME type II (Genbank accession number AE015929) and subsequently described in ACME types II (**Figure 2**) and IV (O’Connor et al., 2018). Many of the ACMEs characterized in the present and previous studies were components of CIs that were separated by multiple DRs, and greatly contributed to the diversity of these MGEs. Interestingly, the *copA* gene and *ars* operon were commonly detected in various distinct positions within or adjacent to ACME types I, II, III, and V. These genes were identified downstream of the ACME type II element in several isolates investigated in the present study (data not shown) but were internalized within the ACME type IIc structures (**Figure 2**). The identification of multiple DRs in common amongst distinct ACME types and the frequent organizational differences observed both within and between each ACME type supports previous studies that suggested ACMEs are assembled in a stepwise, modular fashion (Thurlow et al., 2013). Indeed, ACME diversity was also observed among separate isolates recovered from the oral cavities of the same patient (Supplementary Figure S1) in three cases.

### The Association of ACME With Specific *S. epidermidis* Lineages

Isolates harboring ACME type I investigated here were identified as STs 7 and 17 (**Table 1**), both of which have been previously assigned to GC6 by Bayesian clustering analysis (Thomas et al., 2014; Tolo et al., 2016), a GC that is enriched with ACME-harboring isolates and associated with infection sources. Isolates harboring ACME type II were identified as STs 73 (and ST701, a single locus variant of ST73), 59, 89, 14, and 672, four of which have previously been assigned to GC1, a GC enriched with isolates from non-hospital sources (Thomas et al., 2014; Tolo et al., 2016). Two isolates harboring ACME type IIc from periodontal pockets of two separate patients were identified as ST672, however, this ST was not previously assigned to a GC. All isolates harboring ACME type III were recovered from separate patients and all belonged to ST329, previously associated with GC4. Isolates belonging to GC4 have been associated with a more commensal lifestyle (Thomas et al., 2014). Isolates harboring ACME type IV were identified as STs 153, 297, 130, and 17, of which four were previously assigned to GC6. The isolate harboring ACME type V, identified as ST5, also belonged to GC6.

Distinct ACME types were more commonly associated with isolates belonging to identical or closely related STs, rather than the participant group or sample sites from which each ACME-harboring isolate was recovered. Indeed, many STs identified amongst isolates recovered from the oral rinse samples of healthy participants belonged to GC6 (**Table 2**), a cluster enriched with isolates from infections (Tolo et al., 2016). These findings strongly suggest that the stepwise accumulation of ACMEs occurs in specific lineages of *S. epidermidis*, rather than in specific anatomical sites.

### The Potential Role of ACME in Disease

In the present study, the predominant ACME types detected (II and IV) in *S. epidermidis* harbored the *arc* operon. Researchers have hypothesized that ACME enhances the transmissibility, colonization and persistence of the MRSA USA300 strain on human skin, contributing to the success of this lineage (Diep et al., 2008; Planet et al., 2013). The arginine deaminase pathway encoded by *arc* is responsible for the breakdown of polyamines which are largely L-arginine based. This results in the formation of ornithine, ATP, CO_2_ and ammonia, the latter of which contributes to the internal pH regulation of staphylococci in acidic conditions (Planet et al., 2013; Lindgren et al., 2014). The contribution of the constitutively expressed *arc* operon is likely to be highly advantageous to staphylococcal survival in the acidic environments present in dental plaque. In addition, the ATP generated is likely beneficial to organisms living in nutrient poor, semi-anaerobic environments such as present in periodontal pockets. Polyamines are associated with biological processes such as wound healing and infection clearance and it is therefore likely that they would be highly associated with oral inflammatory diseases such as periodontal disease and peri-implantitis. The *speG* gene, encoding a spermidine acetyltransferase, is thought to mitigate the lethal effects of polyamines on staphylococci. The exact benefit of the *opp3* operon is unclear but encodes an oligopeptide permease ABC transporter (Diep et al., 2006, 2008). The *speG* gene and *opp3* operon were detected in only eight and seven of the 35 ACMEs characterized, respectively, suggesting that these genes are relatively dispensable for *S. epidermidis* in oral environments (McManus et al., 2017; O’Connor et al., 2018).

Interestingly, the *kdp* operon was detected in 12 of the 35 ACMEs characterized by WGS, suggesting that these genes contribute to the survival of *S. epidermidis* in oral environments. This operon encodes a potassium transporter that is important for maintaining intracellular pH homeostasis and metabolic processes in *S. aureus* (Price-Whelan et al., 2013) and likely plays an important role in the adaptation and survival of *S. epidermidis* in dental plaque, which has a significant concentration of K^+^ ions (Margolis and Moreno, 1994).

## Conclusion

This study revealed the significantly high prevalence of *S. epidermidis* in periodontal pockets and subgingival sites of patients with periodontal disease or peri-implantitis, respectively. There was also a very significant difference in the prevalence of *S. epidermidis* harboring ACME in these diseased subgingival sites and periodontal pockets compared to those recovered from healthy subgingival sites and oral rinse samples (**Table 2**). As yet, it is unclear if this organism contributes to disease progression directly. The presence of five main ACME types among oral *S. epidermidis* isolates was identified and extensive genetic diversity among these types was revealed using WGS, which would have been overlooked using previously described multiplex PCRs (Miragaia et al., 2009; Onishi et al., 2013). The *arc* and *kdp* operons harbored by the predominant ACME types identified (II and IV) very likely contribute to the survival of oral *S. epidermidis* under diseased and inflammatory conditions such as periodontal disease and peri-implantitis.

## ETHICS STATEMENT

This study was carried out in accordance with the recommendations of the St. James’s Hospital and Federated Dublin Voluntary Hospitals Joint Research Ethics Committee (JREC) and the Faculty of Health Sciences Ethics Committee of Trinity College Dublin, Ireland. The protocol and all documentation (including consent forms) provided to patients was pre-approved by the Research Ethics Committees. Prior to enrollment in the study, all participants were provided with comprehensive patient information documentation and provided written consent in accordance with the Declaration of Helsinki.

## Author Contributions

AO’ conceived and designed the study, performed the WGS data analysis, and drafted the manuscript. BM conceived and designed the study and helped with the study co-ordination, WGS data analysis, and wrote the manuscript. PK assisted with bioinformatic analyses. GB and TF performed definitive identification of staphylococcal isolates by MALDI_TOF. PC assisted with laboratory processing and isolation of staphylococcal isolates. MOS and IP performed clinical sampling of patients and participants included in the study. DC conceived and designed the study, purchased the required materials, assisted with data analysis, and drafted the manuscript. All authors read and approved the final manuscript.

## Conflict of Interest Statement

The authors declare that the research was conducted in the absence of any commercial or financial relationships that could be construed as a potential conflict of interest.

## Footnotes

1 www.SCCmec.org

2 http://cab.spbu.ru/software/spades/

3 https://blast.ncbi.nlm.nih.gov/Blast.cgi

4 http://sepidermidis.mlst.net/

5 http://www.graphpad.com/quickcalcs/index.cfm

6 https://www.dssresearch.com/KnowledgeCenter/toolkitcalculators/statisticalpowercalculators.aspx
